# A qualitative study to explore women’s perceptions of pregnancy and antenatal care in Chizenga village, Chikwawa, Malawi

**DOI:** 10.1371/journal.pgph.0005515

**Published:** 2026-09-25

**Authors:** Helen Layton, Deborah Nyirenda, Terry Kana

**Affiliations:** 1 Faculty of Education, Liverpool School of Tropical Medicine, Liverpool, United Kingdom; 2 Community Engagement and Bioethics Research Group, Malawi Liverpool Wellcome Research Programme, Blantyre, Malawi; Yale University, UNITED STATES OF AMERICA

## Abstract

National survey data indicate that only around one quarter of women in Malawi attend their first antenatal care (ANC) visit during the first trimester. This study explores women’s perceptions of ANC in Chizenga village, Chikwawa, a rural setting with high maternal mortality and low ANC uptake. Using an interpretivist qualitative design, 14 pregnant women aged 18–44 years were recruited through purposive sampling. Semi-structured interviews were conducted in Chichewa, translated into English, and analysed using thematic analysis to identify patterns across the dataset and develop themes grounded in participants’ accounts. Findings indicate that while women valued ANC and recognised its role in reducing maternal and neonatal mortality, most initiated care after the first trimester. Key themes included understanding of pregnancy health and ANC, nutritional constraints despite awareness, gendered dynamics in ANC attendance, the perceived influence of witchcraft, experiences of respectful and disrespectful care, and structural barriers related to distance and transport. Socioeconomic constraints, particularly distance, transport costs, and limited health literacy, contributed to delayed ANC attendance. Nevertheless, women demonstrated resilience and a strong commitment to maternal health, with some walking over 22 kilometres to access services. Participants voiced a need for increased ANC education and proposed community-based solutions such as health worker outreach and improved access to services. The study calls for context-sensitive policy interventions, including community-based health education, outreach antenatal services, and improved transport support, to ensure equitable access to respectful antenatal care.

## Introduction

Maternal mortality remains a major global health challenge. Its significance is underscored by Sustainable Development Goal 3.1, which aims to reduce the global maternal mortality ratio (MMR) to fewer than 70 deaths per 100,000 live births by 2030 [1,2]. Despite progress, hundreds of women continue to die each day from pregnancy and childbirth related complications, with the vast majority of deaths occurring in low- and middle-income countries, particularly in sub–Saharan Africa [3–5]. In 2020, sub-Saharan Africa accounted for nearly 70% of all maternal deaths worldwide, highlighting the persistent inequities in maternal health outcomes across regions. This disproportionate burden underscores the importance of strengthening access to quality maternal healthcare services, including antenatal care, in resource-constrained settings [6].

Between 2000 and 2015, the global MMR declined from 342 to 223 deaths per 100,000 live births. However, progress has plateaued in many regions since 2016 [7]. As Moyer et al. (2023) note, aggregating data over long periods or across regions can obscure disparities within and between countries, particularly among marginalised populations [8]. Earlier maternal health strategies often prioritised high impact clinical interventions without consistently addressing broader health system and access barriers [5].

More recently, global crises such as the COVID 19 pandemic, climate related disasters, economic shocks, and conflict have further diverted attention and resources from maternal health priorities [9]. In humanitarian settings, maternal mortality rates were more than double the global average despite most deaths being preventable through timely access to quality care [8,10].

Low utilisation of antenatal care (ANC) services remains a significant contributor to poor maternal outcomes in low resource settings. Women in low- and middle-income countries are approximately 23 times more likely to die from pregnancy related complications than those in high income countries [11]. Early initiation of quality ANC, ideally within the first 12 weeks of pregnancy, is essential for reducing maternal and neonatal mortality and for identifying conditions such as anaemia, hypertension, and infections [12].

In response to stalled progress, the World Health Organization (WHO) revised its ANC guidelines in 2016. The previous four visit model, known as Focused Antenatal Care, was replaced with a recommendation for a minimum of eight ANC contacts during pregnancy [12]. These guidelines also emphasise community-based strategies to improve ANC attendance, including partner and community engagement, improved health literacy, and the reduction of barriers to accessing care [13].

Malawi continues to experience a high maternal mortality ratio, estimated at approximately 381 deaths per 100,000 live births, alongside suboptimal uptake of antenatal care [10,14]. ANC often represents a woman’s first point of contact with the health system and provides opportunities to detect complications, promote health behaviours, and link women to other essential health services [15].

Multiple barriers limit antenatal care uptake across sub-Saharan Africa, including in Malawi, where maternal mortality remains high and utilisation of ANC services is often suboptimal [16–18]. Although many of these challenges are shared across the region, their nature and extent vary according to local social, cultural, and health system contexts [19,20]. In Malawi, barriers include long distances to health facilities, poverty, gender inequalities, limited education, and sociocultural practices that influence care seeking behaviour [21]. Poor awareness of ANC benefits, alongside structural and social constraints, influences whether and when women seek care.

Despite increasing facility-based births and growing global attention to respectful maternity care, mistreatment and abuse during childbirth remain widespread, particularly in sub Saharan Africa [22–29]. Evidence suggests that women from rural areas and those experiencing labour complications are more likely to face disrespectful or neglectful treatment from health professionals [30,31]. Contributing factors include poor working conditions, inadequate training, limited supervision of healthcare personnel, and deeply rooted gender norms that limit women’s decision-making power over their own healthcare [32].

In Malawi, the Ministry of Health has recognised these challenges and, through its Safe Motherhood Initiative, has committed to promoting respectful and high-quality maternity care. These efforts include strengthening provider communication skills and addressing systemic challenges that contribute to inequities in service delivery [33–35]. Women’s previous experiences of care strongly influence their willingness to return to facilities and shape community trust in the health system [36].

In Malawi, barriers include long distances to health facilities, poverty, gender inequalities, limited education, and sociocultural practices that influence care seeking behaviour [21]. Distance to health facilities, poverty, competing domestic responsibilities, and shortages of healthcare workers often prevent women from seeking timely ANC, particularly in rural areas where women may walk long distances to reach health centres [37–40]. Women whose partners have higher levels of education are more likely to access skilled ANC services, while household decision-making dynamics further influence maternal health behaviours [6]. Increasing women’s socioeconomic status through improved access to education, income, and information can strengthen autonomy and support informed health decisions [41–47]. Gender norms in Malawi remain deeply rooted in patriarchal structures in which men often act as household decision-makers and gatekeepers of financial and healthcare decisions [48]. Although male involvement has been shown to improve antenatal care attendance and birth preparedness [49,50], local by-laws and sociocultural expectations may also create unintended barriers for vulnerable women seeking care [51]. Together, these interconnected socioeconomic, educational, and gender-related factors influence when and how women access antenatal care in Malawi.

Despite extensive research documenting barriers to antenatal care across sub–Saharan Africa, less attention has been paid to how women in highly localised rural contexts understand and respond to these challenges in their everyday lives. Much of the existing literature focuses on delayed uptake and access barriers, often drawing on large scale surveys or facility-based studies. In hard-to-reach rural settings such as Chizenga village, there remains limited qualitative evidence exploring not only the challenges women face but also their determination to access antenatal care and their ideas for improving engagement within their communities. This study therefore aimed to address these evidence gaps by providing an in-depth, context-specific exploration of pregnant women’s perceptions of pregnancy and antenatal care in Chizenga village, Chikwawa District, Malawi. The findings may help inform community-based maternal health interventions, health education strategies, and policies designed to improve equitable access to antenatal care in rural Malawi.

## Materials and methods

### Ethics statement

Ethical approval for the study was obtained from the Liverpool School of Tropical Medicine (UK) – Protocol Number: GH23(03), the College of Medicine Research and Ethics Committee (COMREC), Malawi – Protocol Number: P.04/23/4064, and the Chikwawa Health Research Coordinating Committee (Malawi). All participants were adult women residing in Chizenga, Malawi. Prior to participation, each woman was provided with a full explanation of the study’s purpose, procedures, voluntary nature, and her right to withdraw at any point without consequence. Written informed consent was obtained from every participant, with each woman either signing or placing a thumbprint on the consent form following the explanation provided in her local language.

Confidentiality and anonymity were rigorously maintained. No personal identifiers were recorded or disclosed, and all data were securely stored in accordance with the ethical and data protection requirements of the Liverpool School of Tropical Medicine, COMREC, and the Chikwawa Health Research Coordinating Committee. Additional information regarding the ethical, cultural, and scientific considerations specific to inclusivity in global research is included in the Supporting Information (S1 Checklist).

### Study design

This qualitative interpretive study was conducted in August 2023 to explore women’s perceptions of pregnancy and antenatal care in Chizenga village, Chikwawa, Malawi. An interpretivist approach was adopted to understand how women perceived and experienced pregnancy and antenatal care within their social and cultural context [52,53].

### Setting

Chizenga village is situated in Chikwawa, a district in the Southern region of Malawi with a population of 564,684. The population of Chizenga village is approximately 1,000. Chikwawa is a malaria-endemic area which is prone to climatic shocks and persistent floods and droughts. It is a hotspot for cholera due to poor water and sanitation systems and practices. The district also has a high incidence of challenges affecting children, including child marriages and neglect, with the prevalence of adolescent pregnancies partly influenced by complex legal and cultural norms [54]. Chikwawa also has one of the lowest attendance rates for antenatal care services in the first trimester of pregnancy (11%) [55]. Barriers to utilising maternal health services in Chikwawa include walking long distances to access health facilities, lack of healthcare personnel, lack of or insufficient items to be used during delivery, long stays, and disrespectful health personnel [55,56].

### Sampling

Purposive sampling was used to select women in their first, second, or third trimester of pregnancy, allowing recruitment of participants with direct experience relevant to the study objectives [57]. Only those who were willing to participate and able to give informed consent were included. Women who were not pregnant, who did not reside in Chizenga village, who were not willing to participate, or who were unable to give informed consent were excluded. Participants were aged 18 years and above in accordance with ethical approval requirements.

Participants were identified through purposive community-based recruitment with the support of local community leaders who were familiar with households in the village. Eligible pregnant women were approached directly in their homes, provided with information about the study, and invited to participate voluntarily. This approach was used to ensure inclusion of women with recent and relevant experience of antenatal care within the rural setting. No eligible participants who were approached for the study declined to participate.

Women were recruited between Friday 28^th^ of July and Thursday 3rd of August 2023. All participants provided informed consent prior to enrolment in the study. In accordance with ethical requirements, informed consent was obtained verbally and subsequently documented through the signing of a consent form. Each participant either signed her name or, where literacy was limited, provided a thumbprint as confirmation of consent. This process was conducted after the Research Assistant (FM) had explained the purpose, procedures, risks, and benefits of the study in detail, ensuring that participants fully understood their involvement. The RA also served as a witness to the consent process, thereby safeguarding the validity and ethical integrity of participant enrolment.

Participants self-selected whether they wished to participate in the study. To enhance variation in perspectives, participants were drawn from across the central catchment area of Chizenga village. Women were purposively selected to reflect a range of experiences, including those at different stages of pregnancy, women who were pregnant for the first time (primigravida) as well as those who had one or more children (multigravida), and women from varied marital statuses and educational backgrounds. This approach helped to ensure that the data reflected the diverse experiences of pregnant women living in Chizenga village.

### Data collection

Fourteen semi-structured interviews were conducted between 28 July and 11 August 2023 in participants’ homes. Following informed written consent, interviews explored women’s understanding of maintaining health during pregnancy and their perceptions of pregnancy and antenatal care.

All of the interviews were conducted in Chichewa and took place in the participant’s home. A topic guide was used to keep the interview focused, using open-ended questions and giving the participants greater freedom to describe and discuss their knowledge and perceptions of pregnancy and antenatal care. The interview topic guide was developed in English and translated into Chichewa by the bilingual RA to ensure linguistic and contextual appropriateness. The RA had prior experience supporting qualitative data collection and received orientation from the principal investigator on the study procedures and ethical requirements before fieldwork commenced.

The interviews were audio recorded using a Dictaphone and detailed notes were taken during or immediately after the interviews. In addition to in-depth interviews, the principal investigator made observational notes during and after the interviews, which captured non-verbal cues, context, and environmental factors. Field notes were recorded to supplement the interview data and provide further insight into participant behaviour, setting, and researcher reflections. The study was designed to centre women’s voices and perspectives. The interview guide used open-ended questions to explore participants' lived experiences.

Interviews typically lasted between 30 and 45 minutes. Efforts were made to conduct interviews in a private space within or near the participant’s home to minimise interruptions and support open discussion. Where other household members were present nearby, care was taken to maintain confidentiality and ensure that participants were comfortable to continue.

At the end of each day, the data from the semi-structured interviews were reviewed to identify and address any gaps, adapt the topic guide and refine the questioning approach based on emerging insights. Refining the research approach involved adjusting how questions were phrased or sequenced to better elicit rich, relevant responses in subsequent interviews. This iterative process also helped improve the overall quality of data collection. The findings were discussed through debriefing sessions between the principal investigator and the research assistant, which allowed the team to capture nuances, clarify meanings, document non-verbal cues, and triangulate data from multiple sources [58].

### Data analysis

All interviews were translated into English by the bilingual research assistant and transcribed verbatim. Data were analysed using thematic analysis, a method for identifying, coding, and reporting patterns or themes within qualitative data [7]. Field notes were reviewed alongside interview transcripts during analysis to support data triangulation and maintain a clear audit trail of analytic decisions. These data contributed to methodological triangulation by integrating interview data, observational field notes, and independent transcript review, thereby supporting a richer and more credible interpretation of the findings.

The principal investigator conducted line-by-line coding of the transcripts to generate initial codes, which were then grouped into broader themes and subthemes. Once themes had been developed, transcripts were exported to ATLAS.ti, a qualitative analysis software, to support further coding, content analysis, and interpretation. ATLAS.ti was used to organise, manage, and retrieve coded data throughout the analysis process. The software supported the systematic comparison of codes across transcripts, helped identify patterns and relationships within the data, and facilitated the development and refinement of themes. This enhanced the transparency and consistency of the analytic process while ensuring that interpretations remained grounded in participants’ accounts. A sample of transcripts was independently reviewed by the research assistant to support consensus-building, minimise bias, and enhance the validity and accuracy of the findings.

Triangulation was used to increase the credibility of the study which involved answering the research question in several ways; interviews, observation, field note analysis, and detailed description of the interpretation process. Quotations from the data were also used to illustrate and support their interpretation.

### Positionality

The principal investigator is a UK-qualified nurse with over twenty years of experience in maternal and child health, including global health roles across sub-Saharan Africa. At the time of the study, the researcher was undertaking postgraduate studies in Global Health and had previously worked in Malawi on maternal health initiatives. While this experience informed the research focus, it also presented a potential for bias.

To minimise bias, reflexivity was employed throughout the research process. The principal investigator remained aware of her position as an external researcher and reflected on how her professional background and outsider status could influence data collection and interpretation. Working alongside a local bilingual research assistant supported cultural sensitivity throughout the study. The Malawian research assistant conducted all interviews in Chichewa and supported translation and transcription. The principal investigator maintained a reflective journal and reflexive field notes to document assumptions, reactions, and observations, thereby enabling critical engagement with the data and helping to ensure that interpretations remained grounded in participants’ accounts.

The study aimed to ensure that the voices and experiences of women living in Chizenga village remained central to the interpretation and presentation of the findings.

### Findings

Fourteen pregnant women aged 18–44 years participated in the study. Most participants were married, although three reported being abandoned by their husbands during pregnancy. Educational attainment was generally low, with participants having either no formal education or primary-level education. The majority were multigravida, while two participants had not yet initiated antenatal care at the time of interview. Most women travelled approximately 11 kilometres to access antenatal services, primarily on foot, reflecting the geographical challenges faced by pregnant women in Chizenga village.

Table 1 outlines the key characteristics of the participants, including marital status, education level, and whether they were primigravida or multigravida.

**Table 1:** Socio-demographic characteristics of the study sample.

ID	Age	Marital Status	Education Level	Gravida	Parity	Gestation in months	Month Initiated ANC	Number of ANC Visits	Distance to Health Facility	Mode of Transport	Accompanied by Husband
1	18 - 20	Married	None	1	0	3	3	1	11 km	Walking	Yes
2	30 - 35	Married	Primary	4	3	3	4	1	11 km	Walking	Yes
3	18 - 20	Married	None	1	0	7	3	4	5 km	Bicycle	Yes
4	18 - 22	Married	Primary	3	2	4	4	1	11 km	Walking	Yes
5	38 - 44	Married	Primary	6	5	6	5	1	11 km	Walking	Yes
6	38 - 44	Married	Primary	8	5	6	5	1	11 km	Walking	Yes
7	20 - 25	Married	None	4	2	8	6	2	11 km	Walking	Yes
8	18 - 22	Abandoned	None	2	1	7	NI	0	–	–	–
9	21 - 25	Married	None	3	1	8	4	3	11 km	Bicycle	Yes
10	30 - 35	Married	Primary	6	5	6	6	1	11 km	Bicycle	No
11	21 - 25	Abandoned	None	3	2	5	NI	0	–	–	–
12	21 - 25	Married	Primary	2	2	9	6	3	11 km	Motorbike	Yes
13	26 - 30	Abandoned	Primary	3	2	8	5	1	11 km	Walking	No
14	18 - 22	Married	Primary	2	1	7	6	1	11 km	Walking	Yes

**NI**: Not Initiated.

**Abandoned**: A number of women reported being married, but were abandoned when their husbands found out they were expecting another child.

This study explored women’s perceptions of pregnancy and antenatal care (ANC) in Chizenga village, Chikwawa, Malawi. Findings are drawn from fourteen semi-structured interviews conducted over a two-week period. Participant characteristics are presented first to provide context for the findings. The results are presented under five key themes that emerged from the data: Pregnancy Health Begins with ANC; Food Knowledge Without Food Security; Accompanied or Alone: Gendered Expectations in ANC Attendance; Respect Shapes Access: Women’s Experiences of Healthcare Worker Attitudes; and Distance, Cost and Determination: Women’s Access to Antenatal Services. These themes reflect the complex social, cultural, and structural factors that shape women’s experiences of pregnancy and their engagement with antenatal care services. Where quotes have been used to understand the voice of the participant, a descriptor has also been provided on marital status, level of education and whether she is pregnant for the first time (primigravida) or is pregnant for at least the second time (multigravida).

### Pregnancy health begins with ANC: What women know and value

The study found that all of the women believed that accessing antenatal care (ANC) was essential to ensuring a healthy pregnancy and safe birth. Participants demonstrated this by describing specific services they expected from ANC visits, such as having their blood pressure, weight, and iron levels checked. Many emphasised the importance of health workers monitoring the baby’s growth and checking for regular movements. Several women also showed knowledge of disease screening during ANC, particularly HIV testing, and spoke about the use of antiretroviral medication to prevent mother-to-child transmission. Others discussed receiving iron tablets, anti-malarial medication, mosquito nets, and access to pain relief. They also identified ANC as a place to receive advice on their pregnancy, particularly antenatal and birth preparedness, and the health of their baby.

‘When we go there (health facility), we will get our weight measured, we stand on the scale. After that we go inside the room and the doctor measures if the baby is kicking or not, after that we get medication that prevents us from malaria. We are also given a mosquito net. We are given HIV screening and vaccinations if we need them.’

(ID 3, Married, no formal education, primigravida)

Overall, the participants’ perceptions of ANC were very positive. All of the women expressed the need to increase education on ANC in Chizenga village to ensure that more women experience a healthy pregnancy and safe birth with skilled health workers at a health facility. However, despite all of the participants understanding the importance of initiating ANC in the first trimester, most women started ANC at around 4 months (16 weeks). All the participants said that they started ANC late due to the long distance they needed to travel to the healthcare facility. Lack of money for transport and food for the journey and husbands not being able to accompany them to their appointment were also reasons the women gave for not attending earlier on in their pregnancy:

‘I have not been to go to an antenatal care appointment yet because I have to go to the hospital so it’s too far. To walk from here (home) to there (healthcare facility) it’s difficult, that’s why I haven’t started yet. I am starting next week. I started antenatal care at four months for my other children too.’

(ID 4, married, primary education, multigravida)

All participants expressed interest in exploring ways to improve awareness and understanding of the benefits of antenatal care (ANC) in Chizenga village. The women suggested that increased health education about ANC, improved access to health facilities and transport, and greater availability of resources could enable more women to access ANC services.

### Food knowledge without food security: Diet

This theme highlights the gap between women’s nutritional knowledge and the harsh economic realities that prevent them from applying it. When the participants were asked what they thought made a pregnancy healthy for both mother and baby, most women talked about the importance of a healthy diet. Several participants also spoke about the food groups healthcare workers had recommended during their ANC appointment.

Several women said they were aware that they should eat more fish, fruit and vegetables; one mother said she had been advised to take vitamin supplements during her previous antenatal appointments. However, most of the women’s responses focused on the fact that they could not vary their diet due to lack of money and resources. Most of the women interviewed said that they lived on Nsima (a thick porridge made out of maize flour and water), beans and cabbage.

Approximately half of the respondents stated that despite having land, they could not grow enough crops to eat regularly and even when it was possible to sell their grain, there was only enough money to eat twice a day. Three participants said that they could only manage one meal and, at times, would go without food for the whole day:

‘Since my husband went to Mozambique, I don’t get any help, so I don’t have any food. Most of the time I don’t eat. You can see (points to cooking pots with no evidence of fire from the morning) since this morning, I didn’t cook anything, so most of the time I feel dizzy because I am hungry and sometimes, I collapse. So, the real problem is how to get food, I don’t have food.’

(ID 11, abandoned, no formal education, multigravida)

### Accompanied or alone: Gendered expectations in ANC attendance

The participants indicated that the village chiefs actively encouraged men’s involvement in caring for their wives during pregnancy. Men were expected to attend ANC appointments with their wives, which made the women feel that their husbands were also being educated and were able to better understand their wives’ needs than if they had not attended ANC.

Most participants reported that their husbands attended antenatal clinics with them, often describing it as a very positive experience. The women explained that healthcare staff encouraged the involvement of husbands in ANC by giving them preferential treatment:

‘If you are with your husband, you receive better treatment and you are seen first. You are brought to the front of the line ahead of the other women who are attending by themselves.

Those without husbands are pushed back and will be the last one to be attended.’

(ID 2, married, primary education, multigravida)

‘We are treated well. When husbands go with their wives they are praised and encouraged to attend each time with their wives so it is a positive experience.’

(ID 4, married, primary education, multigravida)

Several participants also disclosed that they felt significantly more supported when their husbands attended ANC with them. They explained that they felt their husbands better understood their needs, the baby’s development, and the importance of birth preparedness following the appointment. The women also reported that, in previous pregnancies, their husbands had attended the hospital when they went into labour. Although they had not been allowed into the delivery room when their wives gave birth, they were permitted to wait outside and were able to support their wives on the journey home.

Two of the participants said that although their husbands are supportive and will accompany them to ANC, they do not feel comfortable as a man going inside to the appointment. Other women with more than one child said that their husbands were needed at home to care for their other children and could not escort them to the hospital. Despite being married, as these women arrived at the hospital alone, they were treated differently from women attending with their husbands. When asked what happens at the hospital when the women go alone, several women explained:

‘You get the lowest priority.’

(ID 7, married, no formal education, multigravida)

Regardless of marital status, unaccompanied women are kept waiting or ignored ‘My husband is in Mozambique so I walk alone to the hospital. Those who attend with their husbands receive better treatment. I get treated very, very late because the doctors think I’m not married and that maybe the pregnancy is out of wedlock so I am not treated like other married women.’

(ID 8, abandoned, no formal education, multigravida)

The way pregnant women were treated by health workers at the hospital strongly influenced their willingness to attend antenatal care (ANC), with respectful and supportive care encouraging attendance, while perceived discrimination or negative treatment discouraged women from seeking services. Such experiences may therefore act as a barrier to ANC attendance, as some women may choose not to attend in order to avoid similar experiences [23,28].

Although beliefs about witchcraft were not widely discussed, one participant suggested that fears of bewitchment may contribute to delayed disclosure of pregnancy and delayed engagement with antenatal care. She explained:

‘Some women still hide their pregnancy in the early months to avoid being bewitched.’

(ID 5, married, primary education, multigravida)

### Respect shapes access: Women’s experiences of healthcare worker attitudes

Most of the participants in this study described having a positive experience of healthcare workers during ANC appointments at the hospital. The women reported that they felt welcomed and explained that they were treated respectfully during their ANC visits at the healthcare facility. However, there were exceptions, and not all of the participants felt that they had been treated respectfully during their ANC appointment as shown below:

‘If women have forgotten the date of their appointment or you attend on a different date, you get shouted at.’

(ID 3, married, no formal education, primigravida)

Although most women had either experienced or were aware of others who had experienced negative attitudes from healthcare workers during their ANC appointment at the hospital, participants continued to emphasise the importance of attending antenatal care for both maternal and infant health. As one participant explained:

Women who don’t go to antenatal, they have to be advised that it’s better to go because when they go, they get vaccinated, they get tested for different diseases like HIV, if they don’t have enough blood, they are given iron tablets, so it’s better to go there to receive all those things for the goodness of the baby and the mother (ID 5, married, primary education, multigravida).

### Distance, cost and determination: Women’s access to antenatal services

Distance significantly impacted when women accessed ANC as their nearest health facility was 11 km from Chizenga village. Most women reported starting antenatal care late due to the distance they would need to travel to the hospital and lack of transportation. All of the women said that they walked to the hospital and back unless they could borrow a bicycle from someone in the village. Some of the women chose to hire a bike and were taken to their ANC appointment by their husbands. They did this so they did not have to be away from their older children for too long.

Women who could borrow or rent a bicycle and attend their ANC visits with their husbands were able to complete the journey to the health facility and back within five hours. Some participants also mentioned the availability of bicycle taxis, but explained that these were not always reliable or available when needed. Renting a bicycle or hiring a bicycle taxi from Chizenga to the nearest healthcare facility and back cost around 4000 MWK ($3.70), a costly option given that 70% of the population in Malawi lives on less than 2300 MWK ($1.75) a day [59].

‘Borrowing a bike can be expensive for us because when we have a bicycle and it breaks down, we have to pay. The bicycle we used last time broke down three times and we had to repair it. We also have to buy food if we get hungry.’

(ID 3, married, no formal education, primigravida)

Women who were unable to borrow or rent a bicycle reported that the journey to and from the hospital took the entire day, as they had to walk long distances and then wait for hours to be seen, often despite arriving early:

‘I need to start walking at 7 in the morning so that I can come back from the hospital to look after my other children in the afternoon.’

(ID 5, married, primary education, multigravida)

Despite the women’s challenges, all of them were keen to access ANC healthcare despite the long distance to the health centre and hospital. Most of the women felt that the hospital was the only place they could access good advice on their health and the baby’s health. All participants indicated that there was a lack of support for pregnant women in the village, with only two or three saying they would seek advice from their mothers if needed. When asked if all of the women in the village access ANC during pregnancy, most of the women explained that while everyone should attend, there are women who:

‘Don’t want to walk long distances or don’t have money to buy food for the journey, so they stay home.’

(ID 6, married, primary education, multigravida)

The participants believed that some women in the community did not attend ANC due to “laziness” or “ignorance” (ID 12, married, primary education, multigravida). They explained that these women either lacked awareness of the benefits of ANC or failed to prioritise their health and that of the baby. Some participants expressed frustration that, despite the availability of services, there were still women who chose not to attend unless they were already unwell or in labour. Others felt that this behaviour was rooted in limited understanding and called for more education within the community to raise awareness about the importance of ANC:

We need community health workers to educate women to tell them how important it is and what services are offered.’

(ID 2, married, primary education, multigravida)

All of the women spoke openly about the need for community health workers to increase education on the importance of antenatal care (ANC) during pregnancy, and to raise awareness about the services available. They also emphasised the need for improved transport to enable more women to deliver at a health facility. The participants believed that ANC attendance and health-seeking behaviours would improve further if a health facility were established within Chizenga village.

## Discussion

This study explored women’s perceptions of pregnancy and antenatal care in a rural community in Chikwawa District, Malawi. Overall, participants demonstrated a strong awareness of the importance of antenatal care and valued the opportunity to receive health assessments, disease screening, treatment, and health education during pregnancy. Despite this, most women initiated antenatal care after the first trimester, largely due to structural barriers including long travel distances, transport costs, and limited resources. Participants also described the influence of gender norms, experiences of respectful and disrespectful care, food insecurity despite awareness of healthy dietary practices, and the need for greater community-based education about antenatal care. Despite these challenges, women consistently expressed a strong commitment to protecting their own health and that of their babies.

Consistent with previous studies, participants demonstrated an awareness of the benefits of antenatal care during pregnancy, including the importance of diet, monitoring the baby’s growth, monitoring blood pressure and haemoglobin levels, accessing appropriate screening such as HIV, and giving birth at a healthcare facility. However, despite their understanding of the benefits of antenatal care during pregnancy, awareness of the importance of early ANC booking remained limited. Most participants reported initiating ANC at around 16 weeks of gestation [49,53,60,61]. Although Malawi has since adopted the World Health Organization recommendation of a minimum of eight ANC contacts during pregnancy, awareness of ANC guidelines among women in rural communities remains limited. Similarly, findings from previous studies have shown that higher parity is associated with late initiation of ANC [14,51,62]. Delayed uptake remains concerning because it reduces opportunities for the early identification and management of pregnancy-related complications such as postpartum haemorrhage and obstructed labour [14,51,62].

These findings suggest that clearer guidance is needed to support women, particularly those with higher parity, in understanding the importance of initiating antenatal care during the first trimester. Improved counselling during community outreach or early contact with health workers may help address misconceptions and encourage timely attendance. Evidence from a study in South Ethiopia supports this, showing that women who did not receive advice on when to begin ANC were three times more likely to initiate care late compared to those who received such guidance [63].

However, this study found that ANC initiation was not determined solely by parity but by other factors such as the long distance to the healthcare facility, lack of money and food for the journey, and husbands not being able to accompany women to their appointments. These findings are also consistent with findings from previous studies [16,20,21,64–66]. All participants recognised the challenges in accessing ANC and identified how they thought they could overcome some of these challenges. The women expressed a need for community health workers’ presence in Chizenga village. They believed that by improving access to education and explaining the benefits of ANC to women in Chizenga village, more women would make better decisions regarding their health during pregnancy, attend ANC, and choose facility-based care for childbirth.

Evidence shows that interventions involving community health workers can increase knowledge and awareness in rural and underserved communities through community discussions. These approaches also help strengthen links between communities and health facilities, leading to greater utilisation of facility-based maternal care [36]. Community health workers live within the community and therefore have a better understanding of local beliefs and practices that may influence a woman’s knowledge and attitudes toward antenatal care. This insight can help shape strategies to promote early ANC attendance, which is vital for improving maternal and newborn health outcomes [36,67]. One study in rural Malawi found that community health worker interventions can significantly increase the utilisation of ANC [36]. The intervention used community health workers to identify pregnant women and link them to ANC at healthcare facilities. This intervention tripled the proportion of women starting ANC in the first trimester.

Overall, this study found that participants spoke passionately about the importance of attending ANC and giving birth at a healthcare facility. However, several women explained that women also attend because if they do not, they may be punished by village chiefs.

Village chiefs play an important role in encouraging antenatal care and facility-based childbirth. However, while their support has helped increase the number of women attending health facilities with skilled birth attendants [68], some bylaws are enforced in a coercive way, which may negatively affect how women view ANC and giving birth at a facility [39,51]. Several of the participants used the words “encourage” and “advise” when suggesting how to reach out to other women in the village. Evidence suggests that local community members, including family members, neighbours, and women’s groups, play a crucial role in supporting pregnant women. In particular, strengthening women’s groups has been shown to improve ANC uptake and reduce maternal and neonatal mortality in rural settings [31,38]. However, although Aboagye et al. (2023) argue that promoting women’s empowerment programmes without intensive improvements in women’s socioeconomic status would be ineffective, they contend that combining empowerment with active improvements in socioeconomic status can encourage women to improve health-seeking behaviours and ANC uptake in rural communities [40]. Unfortunately, despite how determined the participants were to increase knowledge and awareness of ANC in their community, the women felt they needed support.

In rural Malawian communities, where decision-making often follows hierarchical structures led by chiefs and elders, top-down policy implementation may be more effective than bottom-up empowerment approaches. Walsh et al. (2018) argue that utilitarian, authority-driven models are more aligned with local expectations and social norms, and may therefore be more successful in influencing health behaviours [39]. However, the use of coercive measures, such as imposing penalties on vulnerable women in resource-poor settings, risks reinforcing health, economic, and gender inequities within these rural communities.

### Implications for policy and practice

The findings show that the participants value ANC at a healthcare facility. However, consistent with the literature, their socioeconomic context makes access to ANC challenging. Despite the many challenges, the participants demonstrated remarkable resilience. They expressed a strong desire to support other women in the community and highlighted the importance of improving access to antenatal care through practical, context-specific strategies. These included conducting outreach and sensitisation campaigns, introducing community health workers to strengthen antenatal health education, and increasing social and logistical support for pregnant women at the community level.

The 2016 WHO recommendations on antenatal care for a positive pregnancy experience highlight the importance of community-based interventions to improve communication and support. Furthermore, the recommendations suggest that interventions that improve dialogue around barriers and enablers to utilising ANC services, maintaining health during pregnancy, and increasing support for women and their partners during pregnancy could significantly improve ANC uptake and quality of care [6,67]. Research suggests that eight or more contacts for antenatal care can reduce perinatal deaths by up to eight per thousand births compared to four visits [6]. However, given the number of women who fail to complete four ANC visits in rural communities in Malawi, consideration should be given to how health systems can be strengthened to meet WHO guidance and support more women in accessing ANC.

Until now, women living in rural communities have been expected to walk long distances when they are heavily pregnant, in labour, or after giving birth. The participants in Chizenga village were prepared to walk over twenty-two kilometres to attend ANC, with very little to eat or drink and to a place where they may be treated disrespectfully. This demonstrates how valuable the service is to them and highlights their commitment to improving health outcomes for themselves and their children. Governments should recognise this commitment and respond by ensuring that all women, regardless of socioeconomic, educational, or marital status, have equal access to respectful ANC and the highest attainable health outcomes in pregnancy for both mother and child.

Future strategies to increase the uptake of ANC in rural communities should be designed creatively and be context-specific to reflect the challenges women in resource-poor settings face. Strategies should aim to improve women’s educational and socioeconomic status to reduce inequalities in access to ANC and other maternal healthcare services. Evidence shows that maternal mortality could decrease by up to 70% if all women in sub-Saharan Africa completed primary education [6]. Moreover, women from wealthier households and those with higher educational attainment are more likely to initiate ANC earlier and attend more frequently, highlighting the urgent need to address structural inequalities in maternal healthcare access [6,64,67,69]. Recent studies further corroborate the critical role of women’s education and economic status in ANC utilisation. Adedokun et al. (2022) found that higher educational attainment and improved economic conditions significantly increase the likelihood of adequate ANC service use [70,71]. Similarly, Aboagye et al. and Tessema et al. identified wealth and education as key determinants influencing early initiation and frequency of ANC visits [40,72]. These findings underscore the need to implement strategies that enhance women’s educational and economic opportunities to reduce disparities in maternal health outcomes.

The provision of ANC services could also be expanded to reduce the distance and costs associated with ANC attendance. This could be accomplished by deploying community health workers and midwives to deliver effective and appropriate sensitisation, maternal health campaigns, and mobile clinics in remote communities. Evidence shows that task shifting (delegating elements of maternal healthcare to trained non-physician health workers such as community health workers) can significantly improve access to ANC and continuity of care in low-resource settings [6,36,67]. Community health workers could also help to support and maintain connections between vulnerable populations and healthcare providers.

Healthcare professionals could consider redefining ANC spaces so that men feel more included and the idea that ANC is solely a woman’s domain begins to fade. This study further recommends that health managers ensure staff training and supervision support sustained care and respect for all women attending ANC, regardless of socioeconomic, educational, and marital status.

### Strengths and limitations

This study adds value to the existing literature by showing that positive perceptions of pregnancy and antenatal care from women living in a resource-poor setting exist.

The study shows that women value ANC and want to increase knowledge and awareness of its benefits, not only for themselves but also for other women in their community. A key strength of this study is that data were collected from pregnant women at varying stages of pregnancy, including some who had experienced miscarriage or neonatal loss. These experiences added depth to the findings and contributed to the richness and quality of the data. Using a qualitative approach also allowed the researchers to capture participants’ perceptions and experiences in their natural setting.

Nonetheless, the study had limitations. Firstly, it focused on women over the age of eighteen years. Given the number of adolescent pregnancies reported in Malawi, future studies could also include adolescent women’s perceptions of pregnancy and ANC. Furthermore, the study was conducted in one village and may not reflect the understanding and perceptions of women from other villages in Chikwawa. Finally, only women were included in the study. Given men’s increasing involvement in ANC, future research could explore men’s understanding and perspectives of pregnancy and antenatal care, including the role of village chiefs in influencing care-seeking behaviours. It would also be valuable to include the perspectives of healthcare workers, whose insights could help triangulate the findings and provide a more comprehensive understanding of the barriers and facilitators to ANC uptake in this setting.

## Conclusion

This study demonstrates that, despite considerable barriers, women in Chizenga were determined to access facility-based antenatal care and recognised its importance in ensuring healthy pregnancy outcomes. However, their ability to do so was often constrained by long distances to health facilities, limited financial resources, food insecurity, gender-related expectations, and broader socioeconomic challenges. Despite these barriers, participants demonstrated remarkable resilience, valued antenatal care, and expressed a strong desire to improve maternal health within their community. These findings provide important context-specific evidence from rural Malawi and highlight the need to ensure equitable access to respectful, accessible, and high-quality antenatal care for all women, regardless of socioeconomic, educational, or marital status.

## Supporting information

S1 Checklist Inclusivity in Global Research checklist. (DOCX)
