# Supplementary material for: A qualitative study to explore women’s perceptions of pregnancy and antenatal care in Chizenga village, Chikwawa, Malawi
Source: PLOS Glob Public Health. 2026 Sep 25;6(9):e0005515. doi: 10.1371/journal.pgph.0005515 (PMC13614636; doi:10.1371/journal.pgph.0005515)
Supplement: Supplementary file 1 [file pgph.0005515.s001.docx]

**Ethical considerations, permits and authorship**

**Reported on page number:**
14

**Answer:**
Ethical approval for this study was obtained from the Liverpool School of Tropical Medicine (UK) (Protocol No: GH23(03)), the College of Medicine Research and Ethics Committee (COMREC), Malawi (Protocol No: P.04/23/4064), and the Chikwawa Health Research Coordinating Committee (Malawi).

In addition to formal institutional approvals, permission to conduct the study within Chizenga village was obtained through engagement with local community leaders, who supported community entry and participant identification in line with local governance structures.

All participants provided informed consent prior to participation.

**Reported on page number:**
Methods section (pages 7–15)

**Answer:**
No significant deviations from the approved study protocol occurred during the conduct of this research.

**Answer:**
Yes, this study involved local collaborators.

A Malawian research assistant (RA), fluent in Chichewa and familiar with the local context, played a central role in participant recruitment, informed consent processes, data collection, translation, and transcription.

In-country academic supervision was also provided by a Malawian collaborator affiliated with the Malawi Liverpool Wellcome Trust.

Local collaborators made substantial contributions to the cultural interpretation of findings and the ethical conduct of the study. These contributions are recognised through authorship and acknowledgements in accordance with PLOS authorship criteria.

**Human subjects research**

**Answer:**
Formal ethical approval was obtained through relevant national and institutional ethics committees in Malawi.

At the community level, engagement was conducted with local leaders (including village leadership structures) who act as gatekeepers and representatives of the community. These leaders supported the introduction of the study and facilitated appropriate community entry.

Individual informed consent was obtained from all participants prior to participation.

**Answer:**
The research design was informed by prior experience working in Malawi and engagement with local stakeholders, including in-country collaborators and community leaders.

The involvement of a local research assistant ensured that the study tools, including the interview guide, were culturally appropriate and contextually relevant.

During data collection, an iterative approach was used, allowing emerging insights from participants to shape subsequent interviews. This ensured that the research remained grounded in the lived experiences and priorities of women in the community.

**Answer:**
All participant-facing materials, including the information sheet and consent process, were delivered in Chichewa, the local language.

The research assistant explained the study verbally in accessible, culturally appropriate language, ensuring participants fully understood the purpose, procedures, risks, and voluntary nature of participation.

Participants were given the opportunity to ask questions before consenting. Where literacy was limited, consent was documented using a thumbprint, with the research assistant acting as a witness.

**Answer:**
Findings from this research will be shared with local stakeholders in accessible formats.

This includes:

- Verbal feedback and discussion with community representatives and local collaborators
- Sharing summary findings with in-country partners
- Informing ongoing maternal health initiatives and community-based programmes

The research is also intended to contribute to improving maternal health services and community-based antenatal care strategies in similar rural settings.

**Non-human subjects research**

**Answer:**
N/A

**Answer:**
N/A

**Answer:**
N/A

**Answer:**
N/A

**Answer:**
N/A
